# Blau syndrome: a case report from Palestine

**DOI:** 10.1186/s12969-021-00633-y

**Published:** 2021-08-31

**Authors:** Salam Iriqat, Mohammed Abu Safieh, Manuel Fatouleh, Abdulsalam Alkaiyat

**Affiliations:** 1 Head of Ocular Inflammatory Diseases Head of Clinical Research Uveitis and Medical Retina Consultant, Saint John of Jerusalem Eye Hospital Group, Jerusalem, Palestine; 2 Saint John of Jerusalem Eye Hospital Group, Jerusalem, Palestine; 3 Public Health Department, Faculty of Medicine and Health Sciences, An-Najah National University, P.O.Box:7, Nablus, Palestine

**Keywords:** Blau syndrome, Juvenile idiopathic arthritis, Palestine

## Abstract

**Background:**

This case study documents the first familial case of Blau syndrome (BS) in Palestine characterized with mutation in CARD15/NOD2.

**Case presentation:**

Eighteen years old female was initially misdiagnosed with Juvenile idiopathic arthritis (JIA). The patient had been on steroids and methotrexate treatment for the last 16 years, but did not respond well to treatment. Initial examination at Saint John of Jerusalem Eye Hospital Group clinic showed bilateral intermediate uveitis with camptodactyly. The patient’s sister (aged 19 years) had bilateral intermediate uveitis and camptodactyly. Both eyes of their father had signs of old posterior uveitis. Father’s left eye showed 360 degrees posterior synechia, mature cataract with old Keratic precipitates (KPs). He also had camptodactyly. The patient was referred to pediatric rheumatologist to rule out sarcoidosis.

Lung CT scan showed bronchiectasis, genetic consultation followed. Complete eye examination, full history, refraction, and Optical coherence tomography (oct) were done. Systemic and topical steroid therapy could not control the ocular inflammation. The family then was referred to a geneticist. Genetic analyses showed that the proband and all three family members had an R334q mutation in the CARD15/Nod2 gene.

**Conclusions:**

BS should be considered in the differential diagnosis of childhood uveitis, especially in low and middle income countries where it is misdiagnosed in many cases, which delay appropriate diagnosis and thus control. Genetic analysis of the CARD15/Nod2 gene is helpful in the diagnosis. Steroids alone are not enough to control the disease, other immunosuppressants and biologics are needed.

## Background

Blau syndrome (BS) is a rare autosomal dominant, auto inflammatory granulomatous disease, which is caused by a gain-of-function mutation in the Nucleotide Binding Oligomerization Domain Containing 2 (NOD2) gene [1]. NOD2 gene, which is present on Chromosome 16q12.1 (MIM 186580), encodes for a protein called the nucleotide-binding and oligomerization domain-2 receptor (NOD2-R) which plays an important role in the innate immune system [2]. This receptor is primarily present in the peripheral blood leukocytes and becomes activated when it binds to the muramyl dipeptide (MDP) derived from the bacterial lipopolysaccharides (LPS) and consequently results in the activation of the nuclear factor kappa-light-chain-enhancer of activated B cells (NFKB) protein [3]. This in turn results in the production of inflammatory cytokines and an inflammatory reaction. As a result, the presence of mutations in the NOD2 gene results in the overstimulation of these signaling pathways which ultimately results in the overproduction of inflammatory markers leading to increased presence of granulomatous inflammation. Blau syndrome is characterized with the triad of arthritis, uveitis and dermatitis. These manifestations usually occur in the first few years of life with the skin rash occurring firstly in infancy most of the times [1]; however, patients with BS do not necessary have all of these three manifestations in their lifetime.

On the other hand, Juvenile idiopathic arthritis (JIA), which comes with a similar clinical presentation to BS and is the most common type of arthritis in children [4], is usually one of the top differential diagnoses made for patients with BS.

In this paper we report a female patient with early onset systemic polyarthritis which was initially diagnosed and treated as JIA. She started having eye problems at the age of 7 and was at last diagnosed with intermediate uveitis and consequently BS at the age of 18 along with her sister and father. The rarity of this disease and the fact that this is the first documented familial case of Blau Syndrome in Palestine made it worthwhile to publish.

## Case presentation

An eighteen-year old female was referred to Saint John of Jerusalem Eye Hospital Group uveitis ophthalmology clinic for a specialist consultation regarding bilateral recurrent iridocyclitis. Her long medical history dated back to when she was just 1 year of age when she developed diffuse polyarthritis, mainly targeting both wrists and ankles. Her parents took her to the pediatric rheumatologist who diagnosed her with juvenile idiopathic arthritis (JIA) and treated her mainly with prednisone and methotrexate. During the following years, and because of the lack of compliance to her medications, her arthritis was not well controlled. Therefore, she suffered from intermittent arthritis mainly affecting her wrists and hand joints which were associated with pain and swelling with spontaneous remissions. At the age of 7 years, she started having problems with her eyes; however, she had poor follow up for her condition. She presented multiple times to her local ophthalmologist with recurrent attacks of redness in her eyes and until just 1 year prior to presenting to us, when she was 16 years of age, she was diagnosed with bilateral anterior uveitis.

The ophthalmologist who referred the patient reported her complaint of redness and blurred vision in both eyes since 2 months and on slit lamp examination she had right eye vitreous snowballs and a hazy view along with the presence of anterior chamber inflammatory cells in both eyes but more in the right eye. She also had a history of dry eyes and was on lubricating eye drops.

**Fig. 1:**
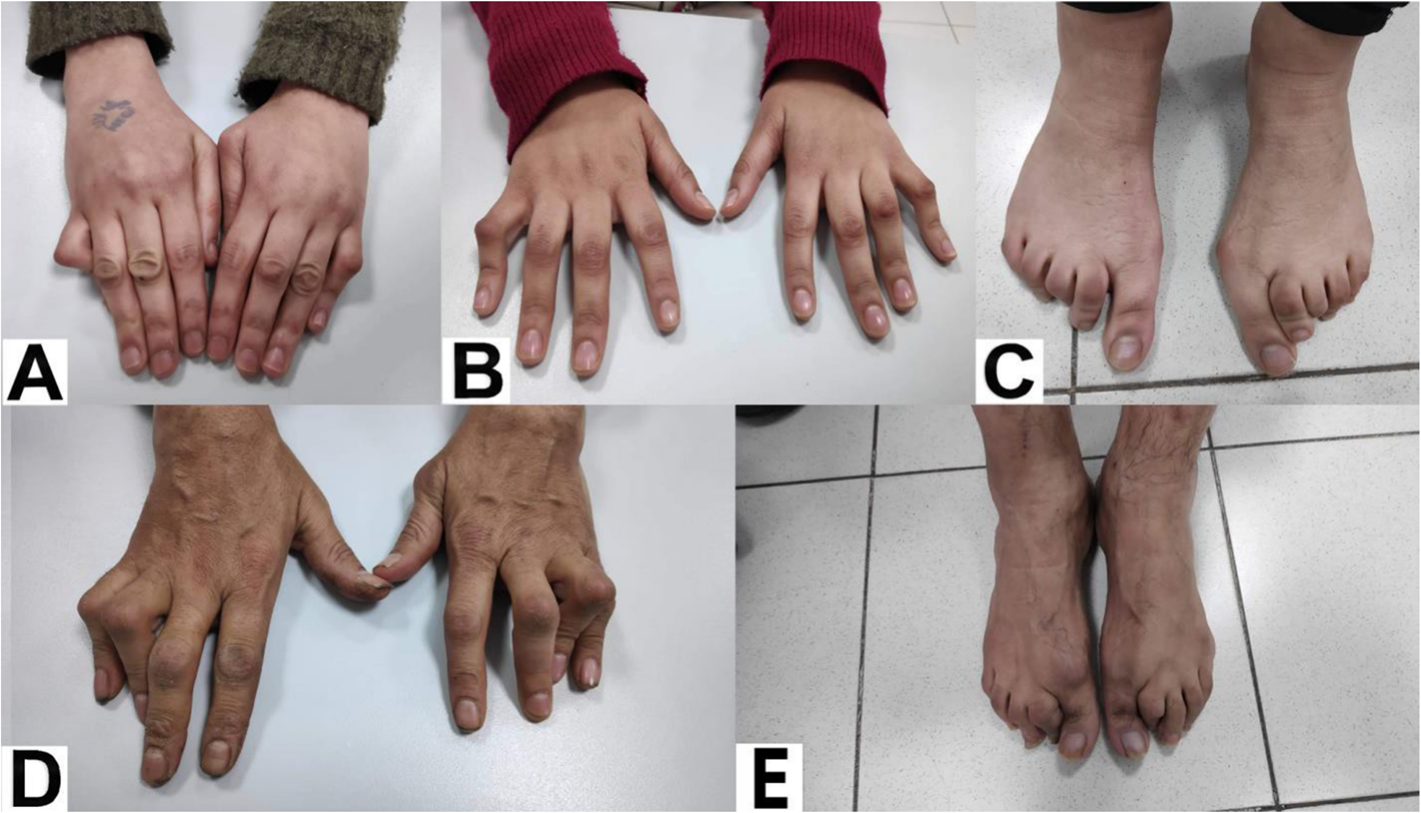
Joint manifestations of Blau syndrome in our patient, her sister and father. **A** Showing our patient with fixed flexion deformity of the proximal interphalangeal joints of the fourth and fifth fingers of both hands. **B** and **C** Showing the patient’s sister and **D** and **E** the patient’s father with residual deformity at the proximal interphalangeal joints of both hands and feet

On presentation to our clinic the patient was on oral prednisolone 5 mg/day, hydroxychloroquine 200 mg/day and omega 3 since 1 month. On slit lamp examination, keratic precipitates and inflammatory cells in the anterior chambers of eyes (+ 3 in the right eye and + 2 in the left eye) were found. Her fundus exam showed vitritis and snowballs bilaterally as well. In addition to the ocular findings, she had painless fixed flexion deformity of the proximal interphalangeal joints of the fourth and fifth fingers in both hands, known as camptodactyly (Fig. 1). Moreover, the patient’s sister, aged 19 years old, had bilateral intermediate uvietis and camptodactyly of hands and feet (Fig. 1). Also both eyes of their father had signs of old posterior uveitis; his left eye showed posterior synechiae 360°, a mature cataract, and old KPs, and he also had camptodactyly of both hands and feet (Fig. 1).

Based on the findings, the patient was diagnosed with recurrent bilateral intermediate uveitis and continued treatment with an increased dose of oral prednisolone (10 mg/day) as well as methorexate and she was given atropine eye drops for 1 week and steroid eye drops which was tapered gradually on each following visit. Subcutaneous Adalimumab (40 mg every 2 weeks) was added shortly after that. Moreover, she was advised to follow up with her pediatric rheumatologist for better control of her arthritis.

Multiple lab tests were ordered and the results were as follows: her complete blood count, kidney and liver function tests, erythrocyte sedimentation rate and C- reactive protein were within normal range. Anti-nuclear antibodies (ANAs) were negative and titers for rheumatoid factor (RF) were within normal range. Serologic testing for Hepatitis B surface antigen (HBsAG) and anti-hepatitis C virus (Anti- HCV) antibodies were negative. She also did the mantoux test (PPD) which was nonreactive. A chest x-ray and a CT scan showed scattered bronchiectasis in both lungs. Pulmonary function test and angiotensin-converting enzyme (ACE) blood levels were also ordered to rule out sarcoidosis and both of them were normal.

**Fig. 2:**
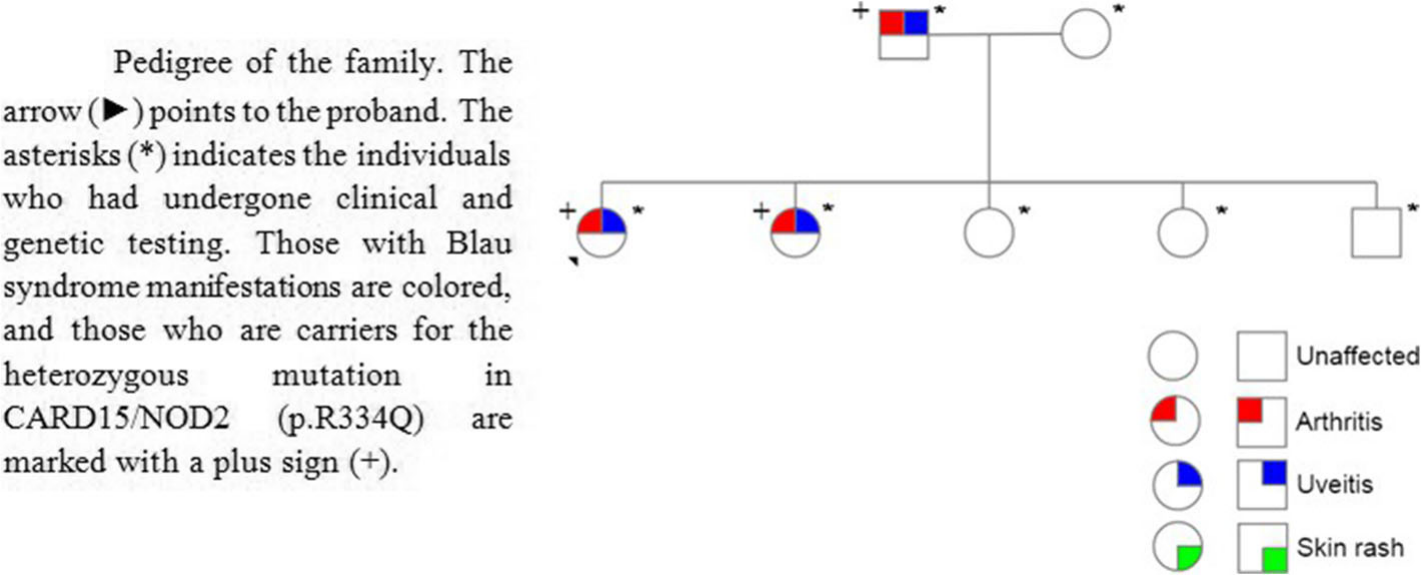
Pedigree of the family. The arrow (►) points to the proband. The asterisks (*) indicates the individuals who had undergone clinical and genetic testing. Those with Blau syndrome manifestations are colored, and those who are carriers for the heterozygous mutation in CARD15/NOD2 (p.R334Q) are marked with a plus sign (+)

In view of the rheumatological presentation and the ophthalmological findings of intermediate uveitis, along with all of the mentioned laboratory tests being normal, it was clear that this patient may in fact not have JIA. The diagnosis was leaning more toward some sort of syndrome. Moreover, due to the fact that not only our patient had these clinical manifestations but also other members of her family, this made us think of a genetic entity. Therefore the next step was to do genetic analysis for the patient and her family via whole exome sequencing which revealed a heterozygous gain-of-function missense mutation in the NOD2 gene (p.R334Q), and therefore Blau syndrome was diagnosed (Fig. 2).

**Fig. 3:**
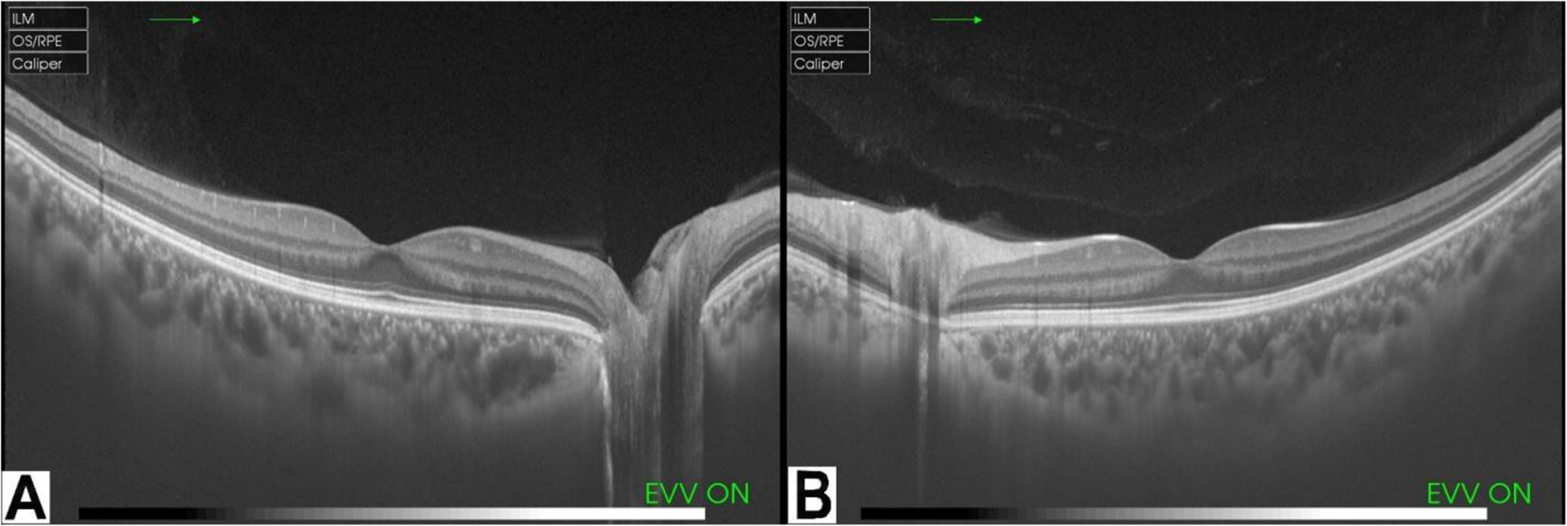
Ocular optical coherence tomograpghy (OCT) with Enhanced Vitreous Visualization (EVV) window of the right eye (**A**) and the left eye (**B**) showing normal retina, normal optic discs, normal macula, and the presence of vitreous changes or opacities (arrows) which are more in the left eye in the index patient with Blau Syndrome

After that, an echocardiogram was done to rule out any cardiac involvement (pericarditis) which was free. Also an ocular optical coherence tomograpghy (OCT) was performed which only showed vitreous changes (Fig. 3). She continued on the same medications with oral prednisolone (10 mg/day) and subcutaneous Adalimumab (40 mg every 2 weeks) as maintenance drugs. She responded good to the treatment and is currently doing well except for mild flare ups which were controlled with steroid eye drops.

## Discussion and conclusions

Pediatric Granulomatous Arthritis constitutes two entities. The sporadic form known as early-onset sarcoidosis, and the familial form known as BS. The familial form was first described in 1985 by Blau [5] and Jabs et al. [6]. Many mutations in the NOD2 gene were described with the two mutations, R334Q and R334W, being responsible for the majority of BS cases [1]. Our patient had the R334Q mutation which is considered one of the common missense mutations of the NOD2 gene that result in BS.

Patients with Blau Syndrome usually present in their early childhood, with dermatitis being the most common first presentation appearing in infancy [1]. The rash has a wide variety of presentations and it usually manifests as a fine eryhtematous maculopapular scaly rash on the trunk and extremities which can sometimes be so mild making it hard for the patient or their family to recall its existence. The only left clue to the presence of the rash is the residual pigmented or scarred skin left by the healing process [1, 7]. Our patient had no history of skin involvement which could be due to the fact that its diagnosis was missed or it did not actually develop similar to other case reports with the incomplete BS clinical triad [7].

Articular involvement mainly manifests as arthritis and tenosynovitis [1]. Arthritis is considered to be the most common clinical manifestation of BS, appearing in the first decade of life [5], as was detected in our patient at the age of 1 year. This arthritis is dominantly polyarticular and most commonly affects the distal joints such as the wrist, ankles, metacarpophalangeal, metatarsophalangeal, and proximal interphalangeal joints [1, 8], and results in joint deformities, such as camptodactyly. Our index case had polyarticular arthritis affecting the distal joints and also presented to us with camptodactyly. Due to the fact that it is a relatively rare disease, diagnosis of BS may not cross the mind when the patient presents with arthritis as the only manifestation early in life as observed in this case. However, this diagnosis may become more of an option when the parts of the clinical triad become more evident along with the presence of a family history and negative anti-nuclear antibody test.

Uveitis constitutes the third clinical manifestation of the triad and is usually the last one to appear in BS patients after dermatitis and arthritis [1]. It has been reported to be diagnosed early on in BS patients with a mean age of onset of 15 months (range 1–84 months) [9], 4.4 years [1], and 5 years [10] in different scientific papers. In this case, the patient had a delayed onset of eye involvement at about 7 years of age and had a definite diagnosis of uveitis at the age of 16. Moreover, similar to the index patient, almost always the uveitis presents bilaterally [10]. Ocular involvement is considered to be the most significant morbidity in BS patients and can result in complications such as, glaucoma, cataracts, chorioretinitis and retinal detachment [8, 10], which makes regular close ophthalmological follow-up crucial to their early detection and treatment.

Other manifestations that were described in BS patients include visceral and vascular involvement such as granulomatous inflammation of the liver and kidney, interstitial nephritis, pericarditis, interstitial lung disease, leukocytoclastic vasculitis, lymphadenopathy and fever [1, 11]. Pulmonary involvement in our patient presented as bronchiectasis on CT scan imaging, which could be as a result of an underlying interstitial lung disease.

As a result of the rarity of BS and the presence of a wide variety of clinical manifestations, there is no one effective treatment for all patients and therefore treatment must be tailored to each and every individual. Treatment choices comprise systemic glucocorticoids, immunosuppressive and biological drugs. Adalimumab is the most common biologic drug used in BS cases and it has been reported to have a good response in treating the uveitis [11]. Index patient responded very well to the addition of adalimumab to her treatment regimen and had remission of the uveitis and improved visual acuity.

This is the first documented case of BS in Palestine. BS remains an under reported entity in the middle-east partly due to its rarity which makes it hard to think of, and secondly due to the fact that the diagnosis of the more common disease JIA, with the considerably similar clinical presentation, is inappropriately given to BS patients. Thirdly, genetic testing and genetic counseling may not be easily accessible in low and middle income countries. It is crucial to detect BS patients early as they will need appropriate and careful management of the uveitis and arthritis to try to decrease morbidity rates.

## Data Availability

The data that support the findings of this study are available from St. John of Jerusalem Eye Hospital but restrictions apply to the availability of these data, which were used under license for the current study, and so are not publicly available. Data are however available from the authors upon reasonable request and with permission of St. John of Jerusalem Eye Hospital.

## Abbreviations

BS: Blau Syndrome
JIA: Juvenile idiopathic arthritis
KPs: Keratic precipitates (KPs).
Oct: Optical coherence tomography
NOD2 gene: Nucleotide Binding Oligomerization Domain Containing 2
NOD2-R: Nucleotide-binding and oligomerization domain-2 receptor
MDP: Muramyl dipeptide
LPS: Lipopolysaccharides
NFKB: Nuclear factor kappa-light-chain-enhancer of activated B cells
ANAs: Anti-nuclear antibodies
RF: Rheumatoid factor
HBsAG: Hepatitis B surface antigen
Anti- HCV: Anti-hepatitis C virus
PPD: Mantoux test
ACE: Angiotensin-converting enzyme
